# Clonal Diversity of ESBL-Producing *Escherichia coli* Isolated from Environmental, Human and Food Samples

**DOI:** 10.3390/ijerph14070676

**Published:** 2017-06-23

**Authors:** Elena Ojer-Usoz, David González, Ana Isabel Vitas

**Affiliations:** Department of Microbiology and Parasitology, University of Navarra, 31080 Pamplona, Spain; dgonzalez@unav.es (D.G.); avitas@unav.es (A.I.V.)

**Keywords:** ESBL-producing *E. coli*, β-lactamase genes, clonal diversity, MLST

## Abstract

This study presents a comprehensive approach of a clonal diversity analysis of 448 Extended-spectrum β-lactamase (ESBL)-producing *E. coli* isolated from environmental, human and food samples in Spain. The phenotypic confirmation of ESBL production was performed by disc diffusion and microdilution methods, while Polymerase Chain Reaction (PCR) and sequencing were used for the molecular characterization of β-lactamase genes (*bla*_CTX-M_, *bla*_SHV_, *bla*_TEM_, *bla*_OXA_). Clonal relationship of isolates was determined by multi-locus sequence typing (MLST). Multidrug resistant strains were present in all the studied niches, with percentages above 50.0%. The most prevalent β-lactamase genes were *bla*_CTXM-14_ (26%) and *bla*_CTXM-1_ (21.4%), followed by *bla*_SHV-12_, *bla*_CTX-M-15_ and *bla*_TEM-42_. MLST isolates were grouped into 26 clonal complexes (CC) and 177 different sequence types (ST) were detected. Despite the high clonal diversity observed, CC10 was the prevalent and the only CC detected in all niches, while other complexes as CC131 were mainly associated to human isolates. The observed prevalence and diversity of these resistant bacteria across the different environments encourages a One Health approach to prevent and control ESBL dissemination between environment and consumers.

## 1. Introduction

The discovery of antibiotics marked a milestone in infectious diseases therapy saving millions of lives. However, antibiotic misuse among humans and animals has led to the emergence of antimicrobial resistance. Extended-spectrum β-lactamase enzymes (ESBLs) are currently considered one of the major public health concerns throughout the world [1]. Among *Enterobacteriaceae, Escherichia coli* is the species that causes the greatest number of infections and has become the main ESBL-producing bacteria. The emergence and wide dissemination of this resistance have important implications in public health due to the risk of clinical treatment failure. Several factors contribute to the spread of ESBLs within and outside of hospitals: the overuse of antibiotics in humans and in food-producing animals [2], agricultural environment [3], food-chain transmission [4,5], water environments [6,7,8] or healthy fecal carriers [9,10]. All these different sources where ESBL bacteria have been isolated were defined as reservoirs that contribute to ESBL transmission. The clinical importance of this resistance is reflected in the number of papers published in the last years that show the increasing prevalence in human isolated *Enterobacteriaceae* of CTX-M enzymes [1,10,11,12], carbapenemases [13,14] and especially the prevalence of CTX-M-15 in Spain and other European regions [10,15]. In fact, a recent publication of the Organization for Economic Co-operation and Development (OECD) regarding antimicrobial resistance places Spain on the top of European countries in both rates of antibiotic consumption and antimicrobial resistance [16].

However, despite the large amount of studies showing the presence of ESBL *Enterobacteriaceae* in different sources, there is a lack of information on integrated studies that give a global perspective of the current situation. In fact, the last report of the World Human Organization on antimicrobial resistance points out the importance of integrating surveillance information that would enable comparison of data from food products, and human and environmental strains [1]. Similarly, the One Health initiative emphasizes the connection of human and animal health to environment as well as the need to improve health in these three domains. Therefore, a collaborative and multidisciplinary approach is necessary for controlling the spread of antibiotic resistance [17].

This study follows World Health Organization (WHO) guidelines and tries to connect data from human, animal and environmental niches. The main objective was to characterize the clonal diversity of ESBL-producing *Escherichia coli* isolated from food products, human samples and different environmental niches in northern Spain in order to gain a better understanding of the diversity and spread pathways of these resistant bacteria.

## 2. Materials and Methods

### 2.1. Sampling and Bacterial Isolation

All ESBL-producing strains included in this study were isolated in Navarra (Spain) from 2009 to 2013. Clinical strains were provided by the Clínica Universidad de Navarra and were collected consecutively from January 2009 to December 2012 as part of the routine diagnostics of the laboratory from different human samples (skin, bound exudate, nasal swab, urethral swab, vaginal and perianal swab, bronchial and ascitic aspirates, deep abscess, sputum, drainage catheter, urine and blood). Ethical approval was obtained from the Ethical Committee Research of the University of Navarra (Plan de Investigación de la Universidad de Navarra Project 024/2012). Food and environmental samples were collected from different locations within Navarra (2010–2012), selected in terms of population (>1000 inhabitants). In addition to the already isolated strains from previous studies [18,19], new sampling was performed over a period of two years (2012–2013) to include a higher variety of food and environmental strains. A total of 580 samples of food were collected including vegetables, cheese, fish, sliced cooked meats and fresh meat products made from beef, poultry and pork. With regard to environmental niches, we have analyzed samples from Waste Water Treatment Plant (WWTP), rivers and farm beds (Table 1).

Resistant strains isolation was performed on ChromID ESBL plates (Biomerieux, Marcy l’Etoile, France), a selective medium containing cefpodoxime. Suspicious resistant bacteria were isolated on nutrient agar (Biolife, Milano, Italy) and strain identification was performed by biochemical tests (API 20E and VITEK^®^, Biomerieux, Marcy l’Etoile, France).

### 2.2. β-Lactamase Characterization

The phenotypic confirmation of ESBL production was performed by combination disk test and double-disk synergy test. The antimicrobial susceptibility to additional antibiotics and minimum inhibitory concentrations were obtained in MicroScan^®^ system (Siemens AG, Munich, Germany). Following Clinical And Laboratory Standards Institute recommendations ESBL-producing isolates were categorized as sensitive and resistant (including “intermediate resistant” and “totally resistant”) [20].

The presence of β-lactamase genes (*bla*_TEM_, *bla*_SHV_, *bla*_OXA_ and *bla*_CTX-M_) was detected by using a modification of two multiplex PCR [21,22]. DNA amplification was performed in a DNA thermal cycler GeneAmp^®^ PCR system 2700 (Applied Biosystems Division, Foster City, CA, USA). In order to identify the *bla* genes a bidirectional DNA sequence analysis was performed by the Macrogen EZ-Seq purification service (Macrogen Europe, Amsterdam, The Netherlands). Each sequence was compared with the sequences included in GenBank and Lahey Clinic web [23].

### 2.3. MLST Analysis

To determinate the clonal dissemination of ESBL-producing *E. coli*, a sequence type analysis was performed following the scheme described by Wirth et al. [24] and seven housekeeping genes were amplified and sequenced for each isolate (*adk*, *fumC*, *gyr*, *icd*, *mdh*, *purA* and *recA*). DNA extraction was performed with a DNeasy^®^ Blood & Tissue kit (Qiagen, Barcelona, Spain) using a pretreatment protocol for Gram-Negative Bacteria. For amplification, 3 μL of DNA extract was mixed with 5 μL of buffer 10× (Bioline, London, UK), 5 μL of dNTPs (Bioline), 1.5 μL of MgCl_2_ 50 mM (Bioline), 2 μL of each primer Sigma-Aldrich, Steinheim, Germany) and 1.5 U of Inmolase™ DNA polymerase (Bioline) in a final volume of 50 μL. The amplification conditions were as follows: 3 min at 94 °C, followed by 30 cycles of 1 min at 95 °C, 1 min at 60 °C (*mdh*, *gyrB*, and *recA*) or 1 min at 64 °C (*fumC*, *icd*, *purA* and *adk*), 2 min at 72 °C, and a final elongation step of 5 min at 72 °C. Sequence reactions were performed by EZ-Seq Service (Macrogen Europe) and sequences data were imported into the *E. coli* MLST database website [25] to determine MLST type. These data were analyzed using BioNumerics v7.5 software (Applied Maths, Sint-Martens-Latem, Belgium).

### 2.4. Statistical Analysis

The results were subjected to statistical processing with the Statistical Package for Social Sciences (SPSS) 15.0 software, (SPSS Inc., Chicago, IL, USA), applying R × C contingency test or Fisher’s exact test depending on the expected frequencies, with a level of significance of *p* < 0.05.

## 3. Results and Discussion

### 3.1. Prevalence in Food and Environmental Samples

Of the 580 food samples analyzed, 19.3% of them were positive for ESBL-producing *Enterobacteriaceae* (ESBL-E). According to results obtained in a previous study [18], fresh meat samples showed the highest prevalence (56.8%), while none of the cheese samples were positive for ESBL-E (Table 1). It must be pointed out that poultry meat carries a high risk of transmission of these resistant strains due to its high presence in this product (89.3%, data not shown). Similar results have been obtained by Skockova et al. [26], who reported high ESBL-E rates in poultry and pork meat in the Czech Republic. With regard to vegetables, only one out of 306 samples were positive for ESBL-producing *E. coli* (0.3%). By contrast, high prevalence of other species were observed (*Enterobacter* spp., *Citrobacter* spp. and *Klebsiella* spp.), coinciding with a report by Reuland et al. [27], who isolated *Enterobacteriaceae* species other than *E. coli* in raw vegetables in similar levels.

Similarly, a wide distribution of ESBL-E throughout the environment was detected because 43.1% of the analyzed samples were positive for these resistant strains. Higher prevalence was observed on WWTP and farms (>50%), while the presence in rivers was lower than the 36.2% reported in a recent study carried out in Switzerland [28].

The high prevalence observed in meat as well as on farms suggests the circulation of ESBL-producing *E. coli* from farm animals to food products, and supports the thesis of resistant bacteria spreading throughout the food chain.

### 3.2. Antimicrobial Resistance

The study of antimicrobial susceptibility points out a high resistance against penicillins, cephalosporins and aztreonam among strains isolated from all niches, combined with co-resistance against quinolones and aminoglycosides (Table 2). In addition, we have observed high susceptibility to carbapenems, coinciding with the results obtained by Egervärn et al. in a study performed on meat imported into Sweden [29]. However, other authors have pointed out the emergence of carbapenem resistance among strains isolated from environmental and clinical samples [13,14,30,31,32].

It must be pointed out that some significant differences regarding antimicrobial resistances in certain antibiotics were observed, depending on the strain origin. For instance, we detected high resistance against cefepime in food and human isolates (94.2% and 94.6%, respectively), while low resistance was observed in farm isolates (2.0%) (*p* = 0.016). Similar low resistances against cefepime were reported in a study performed in meat from Poland [33]. On the other hand, a high resistance to cefoxitin (FOX) was observed in isolates from water environments (rivers and WWTP) when compared to the other isolates. The diversity of species different to *E. coli* found in these environments that usually express AmpC (*Enterobacter* spp., *Citrobacter* spp., *Klebsiella* spp.) could be the reason of this high resistance for FOX. In fact, seven out of 11 isolates from vegetables corresponded to species different from *E. coli*, pointing to irrigation water as the possible origin of contamination as suggested previously. In this paper only *E. coli* isolates have been characterized for the presence of β-lactamase genes. Therefore, further molecular studies in the remaining species should be performed to check the probable AmpC β-lactamase.

Moreover, we detected high susceptibility against aminoglycosides in the strains isolated from all sources, with the exception of amikacin and isolates from food samples (99.5% resistance). Similar results were detected in a study carried out in Sweden [29]. Among food isolates we have not found differences in antimicrobial resistance between poultry, pork and beef meats. For instance, tetracycline resistance differences were not significant (*p* = 0.17) between the three meat origins, consistent with the highly extended use of this antimicrobial in European livestock [34]. Moreover high resistance to tetracycline (83.0%) were observed among food isolates strains coinciding with the study performed by Beninati et al. [35], who detected 85.0% of resistant strains against this antimicrobial.

In contrast, we have detected significant differences (*p* = 0.043) in ciprofloxacin resistance between pork (49.1%), beef (64.3%) and poultry meat (80.4%), which is consistent with the highly extended use of quinolones among poultry farms.

Similarly, clinical isolates showed higher percentage of resistance to quinolones (>70%), in accordance with other European studies [36,37]. Isolates from food also showed high resistance (57.9%) against this antimicrobial family, similar to the data reported by Egervärn et al. [29] Isolates from farm beds showed a percentage of 49.3% of resistance whereas river isolates showed lower percentages.

In addition, a high level of multi-drug resistance (MDR), defined as resistance to three or more structurally unrelated antimicrobial agents, was detected in all niches (Table 2). Food and human isolates showed the highest MDR rates (84.8% and 86.2%, respectively). Similar results were reported by Egervärn et al. [29] regarding food isolates, with levels of multi-resistance between 81 and 98% depending on the type of food. In contrast, Amador et al. [38] observed lower levels of multi-resistance in food in 2009 (31.4%), and lower rates of MDR among human isolates were reported by other authors in 2014 [9,39]. The level of multi-drug resistance observed in strains from other environmental origins was very similar (ranging 60.0%). Similar results were reported by Zurfluh et al. [28] for isolates from rivers and lakes in Switzerland (63.7%). In contrast, Randall et al. [40] found lower levels of MDR *E. coli* isolated from broiler chickens and turkey samples in Great Britain slaughterhouses.

These drug and multi-drug resistance profiles observed in bacteria isolated from food products can be explained by the misuse of antimicrobials in livestock and agricultural production, as well as the movement of resistance bacteria via sewage and manure. These findings pose an emergent health risk through food consumption and show the actual limitations in therapeutic treatment of infections caused by ESBL-producing bacteria.

### 3.3. β-Lactamase Genes

Molecular characterization by PCR and sequencing showed that *bla* genes were present in 75.0% of the strains isolated from food samples, in 86.1% of strains isolated from human samples and in 74.2% of environmental samples. Furthermore, we detected CTX-M-1 and CTX-M-14 producing *E. coli* in all niches (Table 3).

With regard to food isolates, the most prevalent genes were *bla*_CTX-M-14_ (25.0%) and *bla*_CTX-M-1_ (23.9%), followed by *bla*_SHV-12_ and *bla*_TEM-42_. Other *bla* genes such as *bla*_CTX-M-2_ and *bla*_TEM-145_ were present in low percentages. In contrast with other studies [10], encoding *bla*-_CTX-M-15_ genes were detected only in one food sample, despite the fact that this type was the prevalent among clinical isolates. With respect to isolates from poultry meat, *bla*_CTX-M-14_ genes were the most prevalent (34.4%) and a high genotypic variability was observed among these isolates with harbored genes *bla*_CTX-M-1_, *bla*_SHV-133_, *bla*_SHV-5_, *bla*_TEM-145_ and *bla*_TEM-52_. In contrast, Belmar Campos et al. [41] pointed out the prevalence of *bla*_CTX-M-1_ and *bla*_SHV-12_ in poultry meat purchased in Germany. We also detected two multidrug resistant *E. coli* strains with less common phenotypes such as *bla*_CTX-M-8_ and *bla*_CTX-M-2_, which have been observed by other authors in *E. coli*, *Klebsiella pneumoniae* and *Proteus mirabilis* strains [42]. Among isolates from beef meat, the variability was lower and *bla*_CTX-M-14_ genes were also predominant (40%), followed by *bla*_TEM-42_ and *bla*_CTX-M-1_ genes. However, Egervärn et al. [29] detected higher prevalence of *bla*_CTX-M-1_ genes in this meat. Our results showed that 56% of isolates from pork samples harboured *bla*_CTX-M-14_ genes while other authors present pork meat as a reservoir of *bla*_CTX-M-1_ [21]. With respect to the only strain isolated from vegetables, *bla*_CTX-M-1_ was the encoding gene.

In contrast, *bla*_CTX-M-15_ was the prevalent gene among clinical isolates (36.9%), followed by *bla*_CTX-M-14_ (33.8%), *bla*_OXA-1_ (20.0%) and *bla*_TEM-42_ (12.0%). The most common ESBL types characterized in this study (CTX-M-15 and CTX-M-14) were also found in other Spanish studies [43,44] which reported a high prevalence of these genes (30.8% and 27.3%, respectively). Meanwhile, *bla*_CTX-M-1_ genes were present in 11.5% of isolates and only one strain carried *bla*_CTX-M-3_ gene. These results reaffirm the evolution of the prevalence of CTX-M reported years ago by Livermore and Woodford [45] in Spain and detected by other authors more recently [9]. In addition, OXA type enzyme was observed in 26 *E. coli* isolates, and was combined with CTX-M enzyme in 24 isolates.

With regard to environmental isolates, we determined *bla*_CTX-M-1_ as a predominant gene in river samples (37.0%) while *bla*_SHV-12_ was prevalent in farm beds (51.0%). In contrast, other studies reported a high prevalence of *bla*_CTXM-1_ in pig and chicken farms [46,47]. These differences could be due to different antimicrobial treatments and feeds on livestock farms.

### 3.4. Clonal Diversity Study

**Figure 1:**
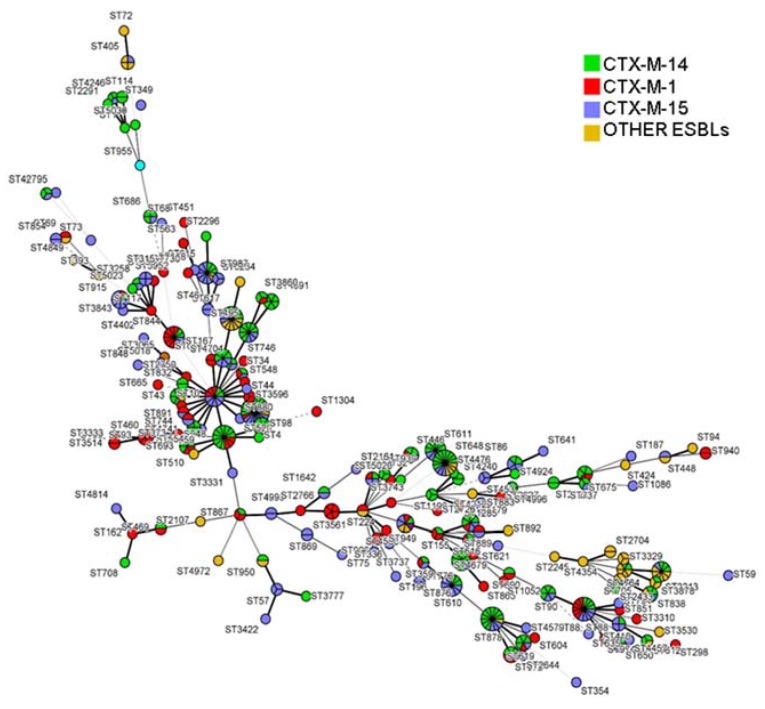
Minimal spanning tree constructed based on the MLST profiles of the 448 ESBL-producing *E. coli* isolates and coloured according to ESBL genes (Bionumerics v7.5, Sint-Martens-Latem, Belgium).

**Figure 2:**
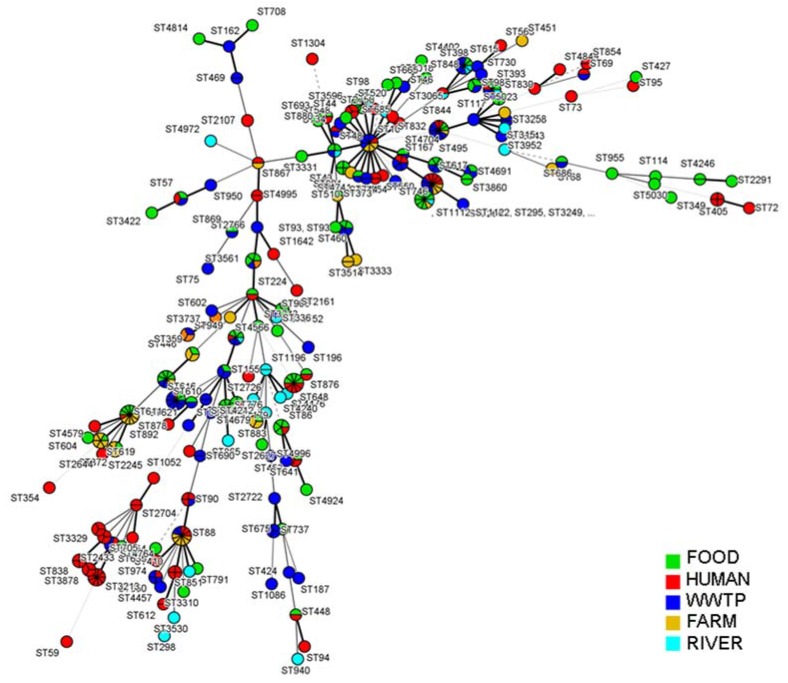
Minimal spanning tree constructed based on the MLST profiles of the 448 ESBL-producing *E. coli* isolates and coloured according samples’ origin (Bionumerics v7.5, Sint-Martens-Latem, Belgium).

MLST analysis of the ESBL-producing *E. coli* isolates (*n* = 448) showed a high clonal diversity regardless of ESBL type (Figure 1) or sample origin (Figure 2). These strains were grouped into 26 different clonal complexes (CC), being CC10 the prevalent clonal complexe in this study (13.3%) and the only one present in all niches (Table 4). It should be noted that CC131 (5.9%) was mainly detected in human samples (*n* = 26). Finally, other complexes were found in three different niches, as CC23 (5.9%) that was present in WWTP, farm and human samples, or CC155 (2.8%) that was detected in food, WWTP and human isolates.

Overall, 177 different sequence types (ST) were detected. Among these, ST98 was the prevalent one (*n* = 17), followed by ST617 (*n* = 13), ST88 (*n* = 12) and ST648 (*n* = 11). It must be pointed out that none of strains have been identified as ST131, however 27 strains from food (*n* = 1) and human samples (*n* = 26) were included in CC131. The remaining isolates were singletons and did not cluster into any CC.

Food isolates belonged to 14CCs and the most extended ST was ST98 (*n* = 13), that was the mainly present in chicken meat (62%), followed by ST776 (*n* = 8) and ST878 (*n* = 6). We have detected ST648, ST776, ST878 and ST98 among the three classes of meat products analyzed in this study, what indicate a high dissemination regardless of meat origin. Furthermore, we have detected five isolates from beef and chicken samples belonging to ST648 and harbouring *bla*_CTX-M-14_ genes. In the same way, Belmar-Campos et al. [41] reported ST648 chicken isolates harbouring *bla*_CTX-M-1_ genes. Similarly, previous studies observed ST648 in ESBL-producing strains isolated from human, poultry and wildlife [48,49,50]. These results point out the spread of this ST among several niches in Europe. Otherwise, isolates harbouring *bla*_CTX-M-14_, *bla*_CTX-M-1_ and *bla*_SHV-12TX-M-14_ belonged to ST359 were detected in chicken meat as well as chicken farms what suggests the existence of a ST359 reservoir among poultry niches. In addition, we have detected only one isolate from pig meat belonged to CC131 (ST2433), traditionally associated to more virulent strains. Finally, other clones as ST57 harbouring *bla*_CTX-M-14_ gene have been detected in different niches (food, WWTP, human samples), what suggests that the dissemination of ESBL genes in *E. coli* may occur through multiple routes including food chain.

WWTP isolates were scattered among 12 CC that included 64STs, the remaining isolates were singletons and did not cluster into any CC. ST611 and ST844 were the most common (*n* = 14), followed by ST615 and ST617 (*n* = 10). ST10 was also a common genotype. Regarding ST611 (CC157), in addition to WWTP environment we have detected this clone among chicken, beef and human samples, associated to SHV-12 and TEM-42 production. Other studies observed this clonal complex among human, poultry and wildlife [51,52]. Moreover, CC10 isolates harbouring *bla*_CTX-M-1_ have been observed in different environments and *bla*_CTX-M-14_ genes belonged to ST10 were isolated from WWTP strains (*n* = 4), farms (*n* = 3) and human samples (*n* = 1). Isolates belonging to ST167 were detected in WWTP (*n* = 3) and human strains (*n* = 2) harbouring *bla*_CTX-M-14_ and *bla*_SHV-12_ genes. In addition, strains from WWTP (*n* = 6) and food samples (*n* = 6) addressed to CC23 (ST88, ST90 y ST650) were associated to CTX-M-14 production. All these results suggest that water systems might act as a reservoir and dissemination route of ESBL resistant genes. Finally, isolates from river samples (*n* = 26) were scattered among 22 different STs and we have detected several STs that were exclusive of river isolates as ST940, ST298 and ST1196.

Regarding farm isolates we have found four CCs, being ST88 the prevalent ST (*n =* 6). All this strains were isolated from poultry farms and were associated to CTX-M-1 (*n* = 5) and SHV-12 production (*n* = 1). Other detected sequences were ST878 (*n* = 5) and ST619 (*n* = 5), associated to SHV-12 production in the first case and SHV-12 and CTX-M-1 production in the second one. We have also detected from a swine farm, a multirresistant strain that belong to ST373 (CC168) and harbour *bla*_CTX-M-1_, *bla*_SHV-12_ and *bla*_TEM-42_ genes.

These results showed the high heterogeneity among farm isolates as well as the risk of transmission to human being through food chain. Similarly, Knapp et al. [53] analyze swine farms and reported high presence of antimicrobial resistant strains and point out this niche as an important reservoir of resistant genes. In addition, we have detected ST648 in chicken, pig and beef meat as well as human samples, what suggest a possible dissemination through food chain.

About 60% of clinical isolates were scattered among 17 CCs, the remaining isolates did not cluster into any CC. A total of 66 different ST were observed, ST3213 was the most common followed by ST88, ST617, ST648 and ST90. We have detected five isolates harbouring *bla*_CTX-M-15_ gene that belonged to ST648 that were also reported by other authors in human and animal samples [49,54].

Moreover, Brisse et al. [55] reported a high diversity among *E. coli* isolated from clinical samples, belonged to 116 STs and 13 CCs. The most common complexes they observed were CC131 (23%), CC10 (15%), CC73 (6%) and CC23 (5%). Similarly, we have detected CC131 (21.6%), CC10 (16.4%), CC23 (16.4%) and CC73 in only one strain (0.9%). According to our study, they also observed a high heterogeneity among CC10 (11 different STs) and CC23 (five different STs). However, Brisse et al. [55] reported high frequency of ST131 meanwhile in our study we have not detected ST131, although we have observed 25 strains from urine samples that belong to CC131 and harbor *bla*_CTX-M-15_ gene. The only strain that harbour *bla*_CTX-M-3_ gene belonged to ST838 (CC131). These isolates belonged to CC131 are interesting because of the high virulence they could present.

We have also detected two strains that belonged to ST393 (CC131) harbouring *bla*_CTX-M-15_ and showing high resistance against quinolones, penicillin, cephalosporin and tetracycline. Other studies reported that strains of this ST were resistant to quinolones and were implicated in extraintestinal infections [56,57].

## 4. Conclusions

In conclusion, this study demonstrates the multidrug resistance and the clonal diversity of ESBL-producing *E. coli* isolated from food, environmental and human samples. The MLST profiles of isolates from food were different from human and environmental isolates and it emphasizes the high diversity of these bacteria throughout these niches. However, the unique CC present in all the aforementioned niches was CC10, while CC131 was mainly associated to human isolates. According to WHO’s recommendations in the ’Global action plan on antimicrobial resistance’ [58], there is a need to strengthen knowledge and evidence through research and surveillance so as to improve our understanding of antimicrobial resistance. Therefore, it is essential to control ESBL-producing *E. coli* spread among animals and environment to minimize human transmission.

## Figures and Tables

**Table 1:** Prevalence of ESBL-producing bacteria in the analyzed samples.

Source	No. of Analyzed Samples	No. of Positive Samples (%)	No. of ESBL-Producing *Enterobacteriaceae*	No. of ESBL-Producing *E. coli*	No. of ESBL-Producing *E. coli* Carrying *bla*_BLEE_
**Food**	**580**	**112 (19.3)**	**196**	**179**	**150**
Fresh meat	169	96 (56.8)	183	175	149
Vegetables	306	14 (4.6)	11	4	1
Fish	37	1 (2.7)	1	0	0
Cooked meat	34	1 (2.7)	1	0	0
Cheese	34	0	0	0	0
**Environment**	**592**	**255 (43.1)**	**252**	**208**	**186**
WWTP	279	163 (58.4)	163	132	117
Rivers	222	45 (20.3)	41	30	26
Farms	91	47 (51.6)	48	46	43
**Human ^a^**	-	-	-	**130**	**112**

^a^ Only *E. coli* isolates were provided by the Clinica Universidad de Navarra.

**Table 2:** Percentages of resistance against different antimicrobials according to isolates origin.

Antibiotic	Origin of Strains				
	Food	WWTP	Rivers	Farms	Human
AMP	100.0	100.0	100.0	100.0	99.2
PIP	99.5	100.0	100.0	100.0	99.2
MZ	98.5	100.0	100.0	100.0	99.2
CZ	99.5	100.0	100.0	100.0	97.7
CXM	99.1	100.0	100.0	100.0	96.9
CPD	99.5	100.0	100.0	100.0	96.2
CTX	97.1	97.9	100.0	100.0	96.2
CAZ	97.6	80.4	83.7	100.0	96.2
FOX	9.7	34.6	43.0	4.0	14.6
FEP	94.2	82.3	18.0	2.0	94.6
AZT	96.6	92.0	87.8	100.0	96.2
AMC	18.0	50.0	36.3	10.0	57.7
AMS	79.1	97.0	92.0	68.0	89.2
TZP	1.9	81.1	20.0	4.0	24.6
ETP	0.0	1.5	2.0	0.0	2.3
MER	0.0	0.0	0.0	0.0	0.8
IMP	0.0	0.0	0.0	0.0	0.0
AK	99.5	0.0	2.0	0.0	8.5
GM	10.7	16.7	8.0	8.0	21.5
TO	7.8	19.5	8.0	10.0	37.7
LV	50.0	38.2	18.0	44.0	70.8
CIP	56.8	41.6	20.0	46.0	72.3
MXF	67.0	52.3	33.0	58.0	75.4
TET	83.0	48.4	71.0	72.0	76.1
TIG	0.5	2.1	2.0	0.0	2.3
COL	21.8	0.0	24.0	10.0	27.7
SXT	40.8	38.0	27.0	34.0	65.4
FOT	2.9	11.9	22.0	2.0	4.6
FM	14.6	26.7	12.0	8.0	10.8
CHL	23.7	13.6	16.3	22.0	33.1
**MDR**	**84.8**	**65.4**	**62.0**	**58.0**	**86.2**

Ampicillin, AMP; piperacillin, PIP; mezlocillin, MZ; cefazolin, CZ; cefuroxime, CXM; cefpodoxime, CPD; cefotaxime, CTX; ceftazidime, CAZ, cefoxitin, FOX; cefepime, FEP; aztreonam, AZT; amoxicillin clavulanic acid, AMC; amoxicillin sulbactam, AMS; piperacillin tazobactam, TZP; ertapenem, ETP; meropenem, MER; imipenem, IMP; Amikacin, AK; gentamicin, GM; tobramycin, TO; levofloxacin, LV; ciprofloxacin, CIP; moxifloxacin, MXF; tetracycline, TET; tigecycline, TIG; colistin, COL; trimethoprim sulfamethoxazole, SXT; fosfomycin, FOT; nitrofurantoin, FM; chloramphenicol, CHL. MDR: multi drug resistance.

**Table 3:** Genotypic characteristics of ESBL-producing *E. coli* isolated from different sources.

Sample Origin	No. of ESBL-Producing	No. of *bla* Genes	Percentages of Detected *bla* Genes (%)				
			_CTX-M-14_	_CTX-M-1_	_CTX-M-15_	_TEM-42_	_SHV-12_
Food	179	150	25.0	23.9	1.2	12.5	18.5
WWTP	132	117	24.5	18.4	11.1	11.7	14.1
Rivers	30	26	17.1	24.4	9.8	4.9	9.8
Farms	46	43	15.5	28.1	21.4	16.7	51.0
Human	130	112	33.5	11.5	39.2	13.1	10.7

**Table 4:** Distribution of main Clonal Complexes (CC) detected in human, environmental, and food samples.

Clonal Complex	Number of Isolates				
	Food	WWTP	River	Farm	Human
CC10	21	16	2	6	16
CC101	2	-	-	5	-
CC131	1	-	-	-	26
CC155	6	6	-	-	1
CC156	2	7	-	-	1
CC168	4	3	-	1	-
CC23	-	7	-	6	13
CC350	1	1	-	-	1
CC398	-	1	-	-	1
CC448	1	-	-	-	2
CC46	2	5	3	-	1
CC648	6	-	-	-	5
CC69	-	1	-	-	1
CC86	5	1	-	-	3

## References

[1] World Health Organization (2014). Antimicrobial Resistance: Global Report on Surveillance. http://www.who.int/drugresistance/documents/surveillancereport/en/.

[2] Angulo F.J., Nargund V.N., Chiller T.C. (2004). Evidence of an association between use of anti-microbial agents in food animals and anti-microbial resistance among bacteria isolated from humans and the human health consequences of such resistance. J. Vet. Med. Ser. B..

[3] Blaak H., Van Hoek A., Veenman C., Van Leeuwen A., Lynch G., Van Overbeek W.M., de Roda Husman A.M. (2014). Extended spectrum beta-lactamase- and constitutively AmpC-producing *Enterobacteriaceae* on fresh produce and in the agricultural environment. Int. J. Food Microbiol..

[4] Zogg A.L., Zurfluh K., Nüesch-Inderbinen M., Stephan R. (2016). Characteristics of ESBL-producing Enterobacteriaceae and Methicillinresistant *Staphylococcus aureus* (MRSA) isolated from Swiss and imported raw poultry meat collected at retail level. Schweiz. Arch. Tierheilkd..

[5] Börjesson S., Ny S., Egervärn M., Bergström J., Rosengren Å., Englund S., Löfmark S., Byfors S. (2016). Limited dissemination of extended-spectrum β-lactamase- and plasmid-encoded AmpC-producing *Escherichia coli* from food and farm animals, Sweden. Emerg. Infect. Dis..

[6] Korzeniewska E., Harnisz M. (2013). Extended-spectrum betalactamase (ESBL)-positive *Enterobacteriaceae* in municipal sewage and their emission to the environment. J. Environ. Manag..

[7] Tissera S., Lee M. (2013). Isolation of extended spectrum b-lactamase (ESBL) producing bacteria from urban surface waters in Malaysia. Malays. J. Med. Sci..

[8] Zhang X.X., Zhang T., Fang H.H. (2009). Antibiotic resistance genes in water environment. Appl. Microbiol. Biotechnol..

[9] Fernández-Reyes M., Vicente D., Gomariz M., Esnal O., Landa J., Onate E., Pérez-Trallero E. (2014). High rate of fecal carriage of extended-spectrum-beta-lactamase-producing *Escherichia coli* in healthy children in Gipuzkoa, Northern Spain. Antimicrob. Agents Chemother..

[10] Livermore D.M., Cantón R., Gniadkowski M., Nordmann P., Rossolini G.M., Arlet G., Ayala J., Coque T.M., Kern-Zdanowicz I., Luzzaro F. (2007). CTX-M: Changing the face of ESBLs in Europe. J. Antimicrob. Chemother..

[11] Ahmed S.F., Ali M.M., Mohamed Z.K., Moussa T.A., Klena J.D. (2014). Fecal carriage of extended-spectrum β-lactamases and AmpC-producing *Escherichia coli* in a Libyan community. Ann. Clin. Microbiol. Antimicrob..

[12] Cantón R., Coque T.M. (2006). The CTX-M b-lactamase pandemic. Curr. Opin. Biotechnol..

[13] Thomas C., Moore L., Elamin N., Doumith M., Zhang J., Maharjan S., Warner M., Perry C., Turton J.F., Johnstone C. (2013). Early (2008–2010) hospital outbreak of *Klebsiella pneumoniae* producing OXA-48 carbapenemase in the UK. Int. J. Antimicrob. Agents.

[14] Pulcrano G., Lula D.V., deLuca C., Roscetto E., Vollaro A., Rossano F., Catania M.R. (2014). Clonal dissemination of *Klebsiella pneumoniae* ST512 carrying blaKPC-3 in a hospital in Southern Italy. APMIS.

[15] Merino I., Shaw E., Horcajada J.P., Cercenado E., Mirelis B., Pallarés M.A., Gómez J., Xercavins M., Martínez-Martínez L., De Cueto M. (2016). CTX-15-H30Rx-ST131 subclone is one of the main causes of healthcare-associated ESBL-producing *Escherichia coli* bacteraemia of urinary origin in Spain. J. Antimicrob. Chemother..

[16] Organization for Economic Co-Operation and Development (OECD) (2015). Antimicrobial Resistance in G7 Countries and Beyond: Economic Issues, Policies and Options for Action.

[17] One Health Initiative. www.onehealthinitiative.com.

[18] Ojer-Usoz E., González D., Vitas A.I., Leiva J., García-Jalón I., Febles A., Escolano M.L. (2012). Prevalence of extended-spectrum β-lactamase-producing *Enterobacteriaceae* in meat products. Meat Sci..

[19] Ojer-Usoz E., González D., García-Jalón I., Vitas A.I. (2014). High dissemination of extended-spectrum β-lactamase-producing *Enterobacteriaceae* in effluents from wastewater treatment plants. Water Res..

[20] Clinical and Laboratory Standards Institute (CLSI) (2012). Performance Standards for Antimicrobial Susceptibility Testing; Twenty-Second Informational Supplement.

[21] Colom K., Pérez J., Alonso R., Fernández-Aranguiz A., Larino E., Cisterna R. (2003). Simple and reliable multiplex PCR assay for detection of *bla*TEM, *bla*SHV and *bla*OXA 1 genes in *Enterobacteriaceae*. FEMS Microbiol. Lett..

[22] Woodford N., Fagan E.J., Ellington M.J. (2006). Multiplex PCR for rapid detection of genes encoding CTX-M extended-spectrum (beta)-lactamases. J. Antimicrob. Chemother..

[23] Lahey Clinic Web, β-Lactamase Classification and Amino Acid Sequences for TEM, SHV and OXA Extended-Spectrum and Inhibitor Resistant Enzymes. http://www.lahey.org/Studies/.

[24] Wirth T., Falush D., Lan R., Colles F., Mensa P., Wieler L.H., Karch H., Reeves P.R., Maiden M.C., Ochman H. (2006). Sex and virulence in *Escherichia coli*: An evolutionary perspective. Mol. Microbiol..

[25] University of Warwick *Escherichia coli* MLST Database. http://mlst.ucc.ie/mlst/dbs/Ecoli.

[26] Skockova A., Kolackova I., Bogdanovicova K., Karpiskova R. (2015). Characteristic and antimicrobial resistance in *Escherichia coli* from retail meats purchased in the Czech Republic. Food Control.

[27] Reuland E.A., Al Naiemi N., Raadsen S.A., Savelkoul P.H.M., Kluytmans J.A.J.W., Vandenbroucke-Grauls C.M.J.E. (2014). Prevalence of ESBL-producing *Enterobacteriaceae* in raw vegetables. Eur. J. Clin. Microbiol..

[28] Zurfluh K., Hächler H., Nüesch-Inderbinen M., Stephan R. (2013). Characteristics of extended spectrum β-lactamase and carbapenemase producing *Enterobacteriaceae* isolates from rivers and lakes in Switzerland. Appl. Environ. Microbiol..

[29] Egervärn M., Börjesson S., Byfors S., Finn M., Kaipe C., Englund S., Lindblad M. (2014). *Escherichia coli* with extended-spectrum beta-lactamases or transferable AmpC betalactamases and *Salmonella* on meat imported into Sweden. Int. J. Food Microbiol..

[30] Isozumi R., Yoshimatsu K., Yamashiro T., Hasebe F., Nguyen B., Ngo T.C., Yasuda S.P., Koma T., Shimizu K., Arikawa J. (2012). blaNDM-1-positive *Klebsiella pneumoniae* from environment, Vietnam. Emerg. Infect. Dis..

[31] Guerra B., Fischer J., Helmuth R. (2014). An emerging public health problem: Acquired carbapenemase-producing microorganisms are present in food-producing animals, their environment, companion animals and wild birds. Vet. Microbiol..

[32] Doyle D., Peirano G., Lascols C., Lloyd T., Church D.L., Pitout J.D.D. (2014). Laboratory detection of *Enterobacteriaceae* that produce carbapenemases. J. Clin. Microbiol..

[33] Wasiński B., Różańska H., Osek J. (2014). Antimicrobial resistance of ESBL and AmpCproducing *Escherichia coli* isolated from meat. Bull. Vet. Inst. Pulawy.

[34] Responsible Use of Medicines in Agriculture Alliance. http://www.ruma.org.uk/.

[35] Beninati C., Reich F., Muscolino D., Giarratana F., Panebianco A., Klein G., Atanassova V. (2015). ESBL-Producing Bacteria and Mrsa Isolated From Poultry and Turkey Products Imported from Italy. Czech. J. Food Sci..

[36] Arnan M., Gudiol C., Calatayud L., Liñares J., Domínguez M.A., Batlle M., Ribera J.M., Carratalà J., Gudiol F. (2011). Risk factors for, and clinical relevance of, faecal extended-spectrum β-lactamase producing *Escherichia coli* (ESBL-EC) carriage in neutropenic patients with haematological malignancies. Eur. J. Clin. Microbiol. Infect. Dis..

[37] Ferech M., Coenen S., Malhotra-Kumar S., Dvorakova K., Hendrickx E., Suetens C., Goossens H. (2006). European surveillance of antimicrobial consumption (ESAC): Outpatient quinolone use in Europe. J. Antimicrob. Chemother..

[38] Amador P., Fernandes R., Prudêncio C., Brito L. (2009). Resistance to beta-lactams in bacteria isolated from different types of Portuguese cheese. Int. J. Mol. Sci..

[39] Gibold L., Robin F., Tan R.N., Delmas J., Bonnet R. (2014). Four-year epidemiological study of extended-spectrum b-lactamase-producing *Enterobacteriaceae* in a French teaching hospital. Clin. Microbiol. Infect..

[40] Randall L.P., Clouting C., Horton R.A., Coldham N.G., Wu G., Clifton-Hadley F.A., Davies R.H., Teale C.J. (2011). Prevalence of *Escherichia coli* carrying extended-spectrum blactamases (CTX-M and TEM-52) from broiler chickens and turkeys in Great Britain between 2006 and 2009. J. Antimicrob. Chemother..

[41] Belmar Campos C., Fenner I., Wiese N., Lensing C., Christner M., Rohde H., Aepfelbacher M., Fenner T., Hentschke M. (2014). Prevalence and genotypes of extended spectrum betalactamases in *Enterobacteriaceae* isolated from human stool and chicken meat in Hamburg, Germany. Int. J. Med. Microbiol..

[42] Casella T., Rodríguez M.M., Takahashi J.T., Ghiglione B., Dropa M., Assunção E., Nogueira M.L., Lincopan N., Gutkind G., Nogueira M.C. (2015). Detection of blaCTX-M-type genes in complex class 1 integrons carried by *Enterobacteriaceae* isolated from retail chicken meat in Brazil. Int. J. Food Microbiol..

[43] Oteo J., Navarro C., Cercenado E., Delgado-Iribarren A., Wilhelmi I., Orden B., García C., Miguelañez S., Pérez-Vázquez M., García-Cobos S. (2006). Spread of *Escherichia coli* strains with high-level cefotaxime and ceftazidime resistance between the community, long-term care facilities, and hospital institutions. J. Clin. Microbiol..

[44] Valverde A., Coque T.M., Sánchez-Moreno M.P., Rollán A., Baquero F., Cantón R. (2004). Dramatic increase in prevalence of faecal carriage of extended-spectrum beta-lactamase producing *Enterobacteriaceae* during non-outbreak situations in Spain. J. Clin. Microbiol..

[45] Livermore D.M., Woodford N. (2006). The beta-lactamase threat in *Enterobacteriaceae*, *Pseudomonas* and *Acinetobacter*. Trends Microbiol..

[46] Hammerum A.M., Larsen J., Andersen V.D., Lester C.H., Skovgaard Skytte T.S., Hansen F., Olsen S.S., Mordhorst H., Skov R.L., Aarestrup F.M. (2014). Characterization of extended-spectrum b-lactamase (ESBL)-producing *Escherichia coli* obtained from Danish pigs, pig farmers and their families from farms with high or no consumption of third- or fourth-generation cephalosporins. J. Antimicrob. Chemother..

[47] Laube H., Friese A., von Salviati C., Guerra B., Roesler U. (2014). Transmission of ESBL/AmpC-producing *Escherichia coli* from broiler chicken farms to surrounding areas. Vet. Microbiol..

[48] Cortes P., Blanc V., Mora A., Dahbi G., Blanco J.E., Blanco M., López C., Andreu A., Navarro F., Alonso M.P. (2010). Isolation and characterization of potentially pathogenic antimicrobial-resistant *Escherichia coli* strains from chicken and pig farms in Spain. Appl. Environ. Microbiol..

[49] Nicolas-Chanoine M.H., Blanco J., Leflon-Guibout V. (2008). Intercontinental emergence of *Escherichia coli* clone O25:H4-ST131 producing CTX-M-15. J. Antimicrob. Chemother..

[50] Guenther S., Grobbel M., Beutlich J., Bethe A., Friedrich N.D., Goedecke A., Lübke-Becker A., Guerra B., Wieler L.H., Ewers C. (2010). CTX-M-15-type extended-spectrum betalactamases-producing *Escherichia coli* from wild birds in Germany. Environ. Microbiol. Rep..

[51] Bonnedahl J., Drobni M., Gauthier-Clerc M., Hernández J., Granholm S., Kayser Y., Melhus A., Kahlmeter G., Waldenström J., Johansson A. (2009). Dissemination of *Escherichia coli* with CTX-M type ESBL between humans and yellow-legged gulls in the south of France. PLoS ONE.

[52] Simoes R.R., Poirel L., Da Costa P.M., Nordmann P. (2010). Seagulls and beaches as reservoirs for multidrug-resistant *Escherichia coli*. Emerg. Infect. Dis..

[53] Knapp C.C.W., Zhang W., Sturm B.S.M., Graham D.W. (2010). Differential fate of erythromycin and beta-lactam resistance genes from swine lagoon waste under different aquatic conditions. Environ. Pollut..

[54] Bonnedahl J., Drobni P., Johansson A., Hernández J., Melhus A., Stedt J., Olsen B., Drobni M. (2010). Characterization, and comparison, of human clinical and black-headed gull (*Larus ridibundus*) extended-spectrum beta-lactamase-producing bacterial isolates from Kalmar, on the southeast coast of Sweden. J. Antimicrob. Chemother..

[55] Brisse S., Diancourt L., Laouenan C., Vigan M., Caro V., Arlet G., Drieux L., Leflon-Guibout V., Mentré F., Jarlier V. (2012). Phylogenetic distribution of CTX-M- and non-extended-spectrum-beta-lactamase producing *Escherichia coli* isolates: Group B2 isolates, except clone ST131, rarely produce CTX-M enzymes. J. Clin. Microbiol..

[56] Blanco J., Mora A., Mamani R., López C., Blanco M., Dahbi G., Herrera A., Blanco J.E., Alonso M.P., García-Garrote F. (2011). National survey of *Escherichia coli* causing extraintestinal infections reveals the spread of drug-resistant clonal groups O25b:H4-B2-ST131, O15:H1-D-ST393 and CGA-D-ST69 with high virulence gene content in Spain. J. Antimicrob. Chemother..

[57] Johnson J.R., Menard M., Johnston B., Kuskowski M.A., Nichol K., Zhanel G.G. (2009). Epidemic clonal groups of *Escherichia coli* as a cause of antimicrobial-resistant urinary tract infections in Canada, 2002 to 2004. Antimicrob. Agents Chemother..

[58] Global Action Plan on Antimicrobial Resistance. http://www.who.int/antimicrobial-resistance/global-action-plan/en/.

